# Spinal manipulation in acute low back pain patients is associated with reduced motor-unit firing variability without altering bilateral coordination: a single-motor-unit EMG study of the lumbar multifidus

**DOI:** 10.1186/s12998-026-00664-w

**Published:** 2026-07-24

**Authors:** Lise R. Lothe, Tim J. L. Raven, Gunnar Sandbæk, Torsten Eken

**Affiliations:** 1 Division of Emergencies and Critical Care, Department of Anaesthesia and Intensive Care Medicine, Oslo University Hospital, Oslo, Norway; 2 Institute of Clinical Medicine, Faculty of Medicine, University of Oslo, Oslo, Norway; 3 Institute of Health and Society, University of Oslo, Oslo, Norway; 4 Division of Radiology and Nuclear Medicine, Department of Radiology, Oslo University Hospital, Oslo, Norway; 5 NEMUS Lillesand AS, Senterveien 30, 4790 Lillesand, Norway

**Keywords:** Low back pain, Muscle tonus, Manipulation, Spinal, Electromyography, Motor neurons

## Abstract

**Background:**

The neurophysiology of high velocity, low amplitude spinal manipulation (HVLA-SM) is poorly understood. We have characterized motor unit (MU) discharge in acute low back pain (ALBP) patients after spinal manipulation and compared with pre-manipulation findings in the same subjects.

**Methods:**

Fine-wire electrodes were implanted bilaterally into the deep lumbar multifidus muscle at the painful spinal level in 9 ALBP patients with pain of less than three weeks duration in March–April 2007. HVLA-SM was applied to the painful spinal level. EMG activity was recorded during manipulation and during spontaneous and voluntary force production while standing and sitting, and individual MUs were identified and their discharge characteristics described. All subjects were compared to their own data recorded before HVLA-SM for effects on gross EMG activity duration, MU discharge rate, interspike interval variability quantified as inter-quartile range for differences between consecutive inter-spike intervals (∆ISI IQR), and common drive coefficient between concurrently active MUs.

**Results:**

No effects on MU discharge rate were evident in the first seconds after HVLA-SM. ∆ISI IQR was reduced by 5.25 ms (95% confidence interval 0.68–9.82; *P* = 0.025) after spinal manipulation compared with pre-manipulation findings, while median MU discharge rate and total duration of gross activity remained unchanged. Notably, the common drive coefficient was lower for bilateral than unilateral MU pairs (− 0.149; 95% confidence interval − 0.087 to − 0.210; *P* < 0.0001). This is in agreement with findings in the same ALBP population before HVLA-SM and in contrast to previous findings during spontaneous standing in pain free subjects, where common drive to uni- and bilateral pairs was not significantly different. MUs showed signs of self-sustained firing, but rotation between units was not observed.

**Conclusions:**

We have established a methodological framework for future investigations using spontaneous muscle activity rather than forced movements to understand the influence of spinal manipulation on the role of the nervous system in postural support. HVLA-SM was associated with reduced MU firing variability, suggesting a shift toward more stable motoneuron output in the deep lumbar multifidus. Longitudinal recovery data are needed to discriminate whether the reduced common drive to bilateral muscle pairs represents a pre-existing trait or a pain-driven state.

## Background

Non-specific low back pain (LBP), in which no definitive nociceptive source can be identified, is the leading cause of disability worldwide [1]. Spinal manipulation is widely used to reduce pain and improve function in LBP and is recommended for both acute and chronic presentations [2]. High-velocity, low-amplitude spinal manipulative therapy (HVLA-SM) is one of the most commonly applied manual interventions [3, 4].

The underlying mechanisms of HVLA-SM remain incompletely understood [5, 6]. Theories have focused on both mechanical and neurophysiological contributions [7]. Proposed mechanisms include disruption of adhesions, increased segmental mobility, stretching and relaxation of hypertonic musculature, release of synovial folds, modulation of alpha-motoneuron activity, enhanced proprioception, and increased pain thresholds via beta-endorphin release [10]. Both a reflex inhibition of hypertonic musculature as well as reflex facilitation of hypotonic musculature after HVLA-SM have been experimentally investigated, with mixed results [8–10]. A comprehensive mechanistic model proposes that the mechanical thrust initiates a cascade of neurophysiological responses that contribute to clinical outcomes [5], underscoring the need to integrate biomechanical parameters with spinal and supraspinal mechanisms, inflammatory mediators, and psychosocial factors such as expectation, fear, and catastrophizing [11].

Motoneuron excitability is strongly influenced by descending neuromodulatory projections from the brainstem. Serotonin and noradrenaline facilitate inward currents, amplifying and prolonging the effects of synaptic input and allowing some motoneurons to continue firing action potentials even after cessation of a brief excitatory stimulus [12, 13]. We have previously suggested that such self-sustained firing may contribute to postural control, including rotation of tonic activity between motor units (MUs) [14–17]. The deep lumbar multifidus (LM), a key stabilizer in spinal postural control, undergoes early pain induced atrophy in LBP [18–22] and is therefore of particular interest. Surface EMG studies of LM are limited by crosstalk and volume conduction [23], and tissue displacement during HVLA-SM precludes the use of needle EMG. Few studies have used intramuscular EMG [24–26] and we are not aware of any intramuscular EMG measurement of spontaneously occurring LM activity after HVLA-SM in common postural tasks such as sitting or standing.

We have developed and validated a method for implanting and verifying the placement of fine-wire EMG electrodes to record from individual MUs in the deep LM [27]. We have shown that there is a strong common drive to MUs both within the same LM and across sides of the spine in healthy individuals [14], whereas patients with acute LBP (ALBP) have reduced bilateral common drive, suggesting altered intermuscular coordination [27].

## Objectives

The aim of this study was to determine in the same ALBP population [27] whether HVLA-SM alters spontaneous and voluntary discharge behaviour of deep LM motoneurons. Specifically, we assessed MU discharge immediately after HVLA-SM, gross-EMG activity and MU discharge rates during prolonged sitting and standing, interspike interval (ISI) variability, common drive between MUs, and evidence of self-sustained firing and MU rotation.

We hypothesised that HVLA-SM would (i) reduce ISI variability, reflecting improved motoneuron firing stability [28–32]; (ii) increase bilateral common drive, indicating enhanced intermuscular coordination [14, 27]; and (iii) facilitate MU rotation, consistent with restoration of normal load-sharing mechanisms.

## Methods

### Study design and setting

The current study was performed during and after HVLA-SM in a cohort of nine acute low back pain patients whose lumbar multifidus single-motor-unit discharge characteristics have been thoroughly examined [27]. The data presented here were obtained in direct continuation during the same experimental sessions, in order to enable comparison between findings before and after HVLA-SM in the same patient. Transition times between the end of pre-SM recordings and the start of post-SM recordings were kept to a minimum, with breaks for the SM procedure not exceeding 10 min. Due to being tethered to the recording equipment by electrode wires, subjects had a restricted range of movement, typically moving only approximately one meter to the treatment table for the side-lying HVLA-SM procedure and back to the sitting/standing area for postural recordings.

Given the novel and invasive nature of recording spontaneous deep LM activity in ALBP patients, this study was designed as an exploratory and hypothesis-generating investigation. Consequently, no single primary outcome was pre-specified, and the analysis aimed to characterize a broad range of neurophysiological parameters to inform future research. All procedures were performed in March–April 2007. This is the first analysis of the post-HVLA-SM dataset. The unique and technically demanding nature of obtaining these invasive recordings justifies the publication of the findings despite the 17-year interval since data collection. We aimed to follow the STROBE statement for cohort studies [33].

### Participants

Subjects with non-specific ALBP of no more than three weeks duration and no overt pathology findings on magnetic resonance imaging (MRI) or physical examination were recruited to the study from two multi-disciplinary private outpatient clinics. Patients with contained intervertebral disc protrusion or small prolapse without neurological findings were accepted. A clinical neurological screening was performed ensuring that there was no neuropathology. Back function and spinal movements were assessed, range of motion was evaluated in three planes, and level of pain was determined by palpation [27].

### Electrode implantation

An experienced interventional radiologist implanted custom-made fine-wire EMG electrode bundles bilaterally at the symptomatic spinal level under computer tomography (CT) guidance. The electrode bundles were made from three or four 25 cm long, 50 µm diameter Teflon-insulated platinum/iridium wires (A-M Systems, Everett, WA) that were twisted tightly together. The distal 1–2 mm was bent back 180° creating a hook and cut transversely to expose a recording surface area of 0.002 mm^2^ per wire. The skin was disinfected and anaesthetised with a small volume of lidocaine injected subcutaneously [27]. The sterilised electrodes were fed through an 18G epidural needle (Portex System 1 Epidural Minipack, Smiths Medical ASD, Inc., Keene, NH) that was removed after implantation. A low radiation computer tomography (CT) scan was obtained before and after positioning of the needle, and a final scan to document initial electrode position was taken after the subject had moved around for the electrodes to settle in the tissues. Each scan had an estimated radiation exposure of 0.1 mSv, equivalent to approximately one week of natural background radiation. The electrodes were fastened to the skin by sterile reinforced adhesive skin closures (Steri-Strip R1546, 3 M Health Care, St. Paul, MN) and protected with a transparent film dressing (Tegaderm Film 1626W, 3 M Health Care, St. Paul, MN). Finger to floor distance during full lumbar flexion was measured before and after implantation of the electrode bundle to ensure that the implant did not hinder lumbar movement.

In order to record from a particular lumbar segment the electrodes had to be inserted deeply into a region close to the inferior half of the spinous process [34, 35], as only the deepest fascicles of the LM are believed to be uni-segmentally innervated [36]. Comparison of needle placement and fine-wire EMG electrode positioning was performed by measurement on the CT images [27, 37]. The desired placement was defined as the inner 1/3 of the paraspinal bulk. Thirteen electrodes were successfully positioned on target [27].

Our setup did not attempt to quantify any long-term changes in pain or motor output as it is not feasible to keep the electrode embedded in the muscle for more than a few hours without incurring the risk of infection and compromised integrity of the electrode.

### Spinal manipulation

An HVLA-SM procedure was applied bilaterally in the lateral recumbent position to the facet joints at the level of the painful segment. Due to its deep location and proximity to the vertebral bodies the multifidus has a faster response than the erector spinae to HVLA-SM; the up-side musculature also has a faster response and is likely stretched more due to the rotational nature of the side-lying manipulation [25]. A finger-pull contact on the spinous process at the level of electrode implantation was the preferred manipulative technique in order to minimize the risk of manual disruption of the recording equipment. Most subjects were placed in the left recumbent side-lying position first. The manual force applied to the spine in such a procedure is typically delivered in approximately 200 ms, with linear vertebral displacements less than 10 mm after a brief preload phase [38]. The HVLA-SM procedure was performed by the same experienced chiropractor in all experiments. The chiropractor was connected to signal ground while performing the procedure.

### EMG recording

All EMG recording and signal processing procedures were conducted in adherence to the standards for reporting EMG data endorsed by the International Society of Electrophysiology and Kinesiology (ISEK) [27, 39]. Differential recordings were made from the electrode pair in the bundle that provided the best possible discrimination of MU activity. The EMG signals were fed through an amplifier with band pass filtering at 20 Hz–25 kHz and a 50 Hz notch filter, digitized at 50 kHz per channel, and stored on a computer with concurrent video recordings of the experiment [27]. A standard ECG pad was placed in the midline and used as a ground reference electrode.

Spontaneously occurring tonic activity was recorded before and after HVLA-SM, during sitting without back support with hips and knees bent at 90 degrees and when standing in an unsupported, natural posture [27]. The subject was watching a movie for 10–20 min in each position. During this part of the experiment, the subject did not receive any feedback from the equipment or staff. Voluntary tonic activity for periods of 10–30 s was obtained by asking the subject to deliberately regulate the force of contraction of the LM by leaning slightly forward at the hips and listening to the MU action potential (MUAP) discharges through speakers [14, 27].

Periods of spontaneous and voluntary behaviour, sitting and standing, and HVLA-SM were identified from the video recordings and documented in the project database. The EMG activity during a 5 s period immediately before HVLA-SM was compared to a 5 s period immediately following HVLA-SM while the subject was still on the treatment table in a side lying position. The total recording time was defined as the time recorded while the subject was quietly standing or sitting while watching a movie without interference, or performing experimenter-controlled voluntary movements. A crude measure of gross activity duration was obtained from the differentially recorded raw signal [27]. Periods of electrical noise were later excluded from analysis.

### EMG analysis

Spike2 version 7 (Cambridge Electronic Design, Cambridge, UK) standard detection and sorting algorithms were utilized offline to identify MUs and to discriminate them from nearby units [27]. Instances where Spike2 failed to correctly identify MUAPs were manually controlled and corrected. The time stamp of each MUAP was stored together with unique subject and MU identifiers in a custom-built database (FileMaker Pro 11.0v3, FileMaker Inc, Santa Clara, CA) from where ISIs, instantaneous discharge rates, and train lengths were calculated.

A MUAP train was defined as a succession of single-motor-unit action potentials where all ISIs were shorter than 500 ms, from this discharge rate was determined. Interval-to-interval MU discharge variability was assessed by computing the difference between successive interspike intervals (ΔISI) and reporting the inter-quartile range (IQR) of the resulting distribution [27, 40]. MU discharge episodes with repetitive doublets (ISI 2.5–20 ms) were excluded from ∆ISI IQR analysis [14, 27, 41]. Only ISIs from 4 to 500 ms (i.e., discharge rates from 250 to 2 pulses per second [pps]) in MUs with at least 25 ISIs were included in discharge rate analyses.

Common drive to the motoneuron pool was assessed by determining the extent of cross correlation between concurrently active motor units [27, 42, 43]. All computations were performed offline in Spike2. Following MU discrimination, a continuous waveform representing smoothed discharge rate was calculated by replacing each discharge event with a 600 ms wide raised cosine waveform of unit area symmetrical about the event time. A second-order high-pass Butterworth filter with cut off frequency at 0.75 Hz was then applied to remove mean discharge rate and low-frequency oscillations. The common drive coefficient (CDC) was defined as the maximum value within 0 ± 50 ms of the cross correlogram between high-pass filtered smoothed frequency traces from a MU pair to capture neural transmission delays up to ≈50 ms while excluding long-range slow co-variation [43]. Individual cross correlograms from the same MU pair were computed as a weighted average using recording duration as weight. This produced a single resultant correlogram for each MU pair in each recording situation [27]. Minimum duration for a trace was 5 s.

Pairs of concurrently active MUs were examined for evidence of self-sustained firing, i.e., where a MU (test unit) was recruited from inactivity to its preferred firing range or was abruptly derecruited while the discharge rate of one or more units which were already active (reference units) remained unchanged [17, 27, 44, 45]. Rotation of activity was defined as occurring when a test unit was recruited from inactivity to stable tonic activity while a reference unit continued to fire with little or no change in discharge rate, and the reference unit subsequently was derecruited while the more recently recruited test unit continued to fire with little or no change in discharge rate [14, 27].

### Statistical methods

Statistical analyses were carried out using JMP 11.0.0 (SAS Institute Inc., Cary, NC). All results are given as medians with 25th and 75th percentiles unless otherwise specified. Coefficients in regression models are reported with 95% confidence intervals (CI). A two-tailed *P* value of < 0.05 was considered statistically significant. For comparison between proportions of CDCs in the higher-CDC sub-distribution (Fig. 7), non-overlapping 95% CIs were regarded as statistically significant differences.

Linear mixed models using the Restricted Maximum Likelihood (REML) fitting method were utilized to test or control for the effects of a number of predefined independent variables (age, sex, daily coffee intake [46], whether the electrode was implanted on the side of the subject’s dominant hand, whether the electrode was on a painful or non-painful side, and whether the electrode was positioned “on target” in the inner third of the LM) on total gross EMG activity duration, median MU discharge rate, ∆ISI IQR, and CDC. Pain duration was not used as a covariate in the mixed model. Only independent variables with a *P* value < 0.10 in univariate analysis were eligible for the models. This statistical modeling strategy, including the subsequent backward elimination of non-significant covariates, was pre-specified in the study protocol to ensure a systematic and unbiased approach to model construction. In accordance with the experimental design, before vs. after HVLA-SM, spontaneous versus voluntary activation, sitting vs. standing, unilateral vs. bilateral MU pair (for CDC), and median MU interspike interval (for ∆ISI IQR) were always entered as independent variables in the multivariate model that was tested. Subject, side (left/right) nested under subject, as well as MU nested under subject and side were entered as random effects to account for repeated measures and hierarchical data structure. No specific covariate for elapsed session time was included in the model, as the study prioritized the immediate and sustained effects of the manipulation within a single experimental session. For CDC analyses, left/right side was not part of the model, as this would preclude analyses of unilateral vs. bilateral MU pairs. Similarly, MU and spontaneous vs. voluntary activation were not part of the model for gross EMG activity duration. The pre- and post-HVLA-SM MU samples are non-overlapping at the MU level; subject and side are the within-subject pairing units, and the analysis is therefore between MU samples drawn from the same subject and side before and after. Missing data were not imputed. Variables in the final model were selected through backward elimination: After each run, the single least significant independent variable was removed, and the model was re-run until only variables with *P* < 0.05 remained. The proportion of variation in the response attributed to the model rather than random error is reported as RSquare.

## Results

### Participants

A convenience sample of thirteen ALBP patients from two private outpatient clinics were screened for participation [27]. Two males were excluded due to pathology findings on MRI. Eleven subjects with nonspecific ALBP were included. Data was lost in one female subject due to technical problems and EMG recording was unattainable in one female subject due to no electrical signal at the electrode tip, possibly caused by extensive fatty infiltration in the target muscle. Data was recorded from the remaining nine ALBP subjects (Table 1) with an episode of no more than 3 weeks duration (median duration 2 days, range 1–21 days), palpation findings of lumbar spine tenderness, no leg pain, no neurological complications, and no overt pathology findings on MRI examination. Median pain intensity was 6 on an 11-point numerical rating scale (range 2–7), unilateral pain was reported in 4 subjects and bilateral pain in 5 subjects. Pain was not re-measured post-SM since this experimental setup aimed to measure objective neurophysiologic changes and not subjective pain changes. One subject had only one electrode successfully implanted, and one had unacceptably poor recording quality on one side. Thus, recordings from 16 electrodes were available for analysis.

**Table 1:** Statistics, grouped data

	Total	Spontaneous sitting	Spontaneous standing	Voluntary standing
**Subjects**				
Number (m:f)	9 (8:1)			
Age (years) (median; range)	38 (26–59)			
Dominant side (right:left:missing)	7:1:1			
Coffee consumption (cups/day) (median; range)	3 (0–8)			
Electrodes recorded from (on target: off target)	16 (13:3)			
Recording time (minutes) (sum; range)	241 (19–37)	119 (9–19)	114 (5–20)	9 (0.5–2.4)
**Gross muscle activity**				
Gross activity recordings, subjects and sides, total material (n)	16	16	16	16
Duration of gross activity, subjects and sides, total material (min)	194	79	100	15
Duration of gross activity, per subject and side (s) (median; range)		169 (0–842)	369 (0–972)	43 (28–138)
Duration of gross activity, per subject and side (% of recording time; range)		22 (0–99)	56 (0–100)	98 (90–100)
Single-unit activity within spike trains*				
Units, total material (n) (on target: off target)†	69 (62:7)	24 (24:0)	29 (27:2)	28 (23:5)
Duration of analyzed single-unit activity, total material (minutes)	164	75	78	11
Duration of analyzed single-unit activity, per unit (s)		159 (53–278)	148 (78–253)	22 (17–30)
Duration of analyzed single-unit activity, per unit (% of recording time)		15 (7–27)	24 (8–31)	34 (27–85)
Intervals, total material (n)	59,168	25,403	29,098	4667
Intervals, per unit (n)		1026 (248–1571)	943 (479–1459)	142 (105–252)
Median discharge rate, per unit (pps)		6.01 (5.09–6.33)	6.76 (5.84–7.99)	7.07 (5.86–8.40)
Mean discharge rate, per unit (pps)		5.92 (5.12–6.66)	6.81 (5.84–8.05)	7.24 (5.79–8.76)
Interquartile range for difference between successive interspike intervals (∆ISI IQR) (ms)		34.4 (30.8–43.6)	45.6 (39.2–55.7)	24.3 (18.9–28.9)
Coefficient of variation for interspike intervals, per unit (%)		24.4 (22.0–31.3)	34.2 (30.1–37.4)	19.0 (15.6–25.9)
**Common drive coefficients**				
Unit pairs, unilateral (n)‡	49	16	21	14
Unit pairs, bilateral (n)	55	9	29	19
CDC, unilateral		0.23 (0.14–0.34)	0.20 (0.11–0.40)	0.35 (0.14–0.47)
CDC, bilateral		0.09 (0.03–0.16)	0.10 (0.00–0.23)	0.15 (0.07–0.25)

Numbers are medians and quartiles unless otherwise specified

^*^Spike trains are defined as consecutive spikes with intervals < 500 ms. All analyses of firing frequencies and ∆ISI IQR are in trains with ≥ 25 intervals; discharge rates higher than 250 pulses per second (pps) are excluded

^†^12 units were recorded from in more than one task: 4 in spontaneous sitting and spontaneous standing; 4 in spontaneous standing and voluntary standing; 4 in voluntary standing and spontaneous sitting. No unit was recorded from in all three tasks

^‡^2 unit pairs were recorded from in more than one task: 1 in spontaneous sitting and spontaneous standing; 1 in spontaneous standing and voluntary standing. No unit was recorded from in all three tasks

2 unit pairs were recorded from in more than one task: 1 in spontaneous sitting and spontaneous standing; 1 in spontaneous standing and voluntary standing. No unit was recorded from in all three tasks

Finger to floor distance remained unchanged for all subjects before and after implantation, confirming that the electrode did not impede movement. Each trial lasted 2–4 h including the time period before HVLA-SM, and visual inspection after removal verified that all electrode bundles were undamaged. Room temperature varied from 21 to 23 °C between different experiment days but there was no variation during an individual experiment.

### Immediate response to spinal manipulation

**Fig. 1:**
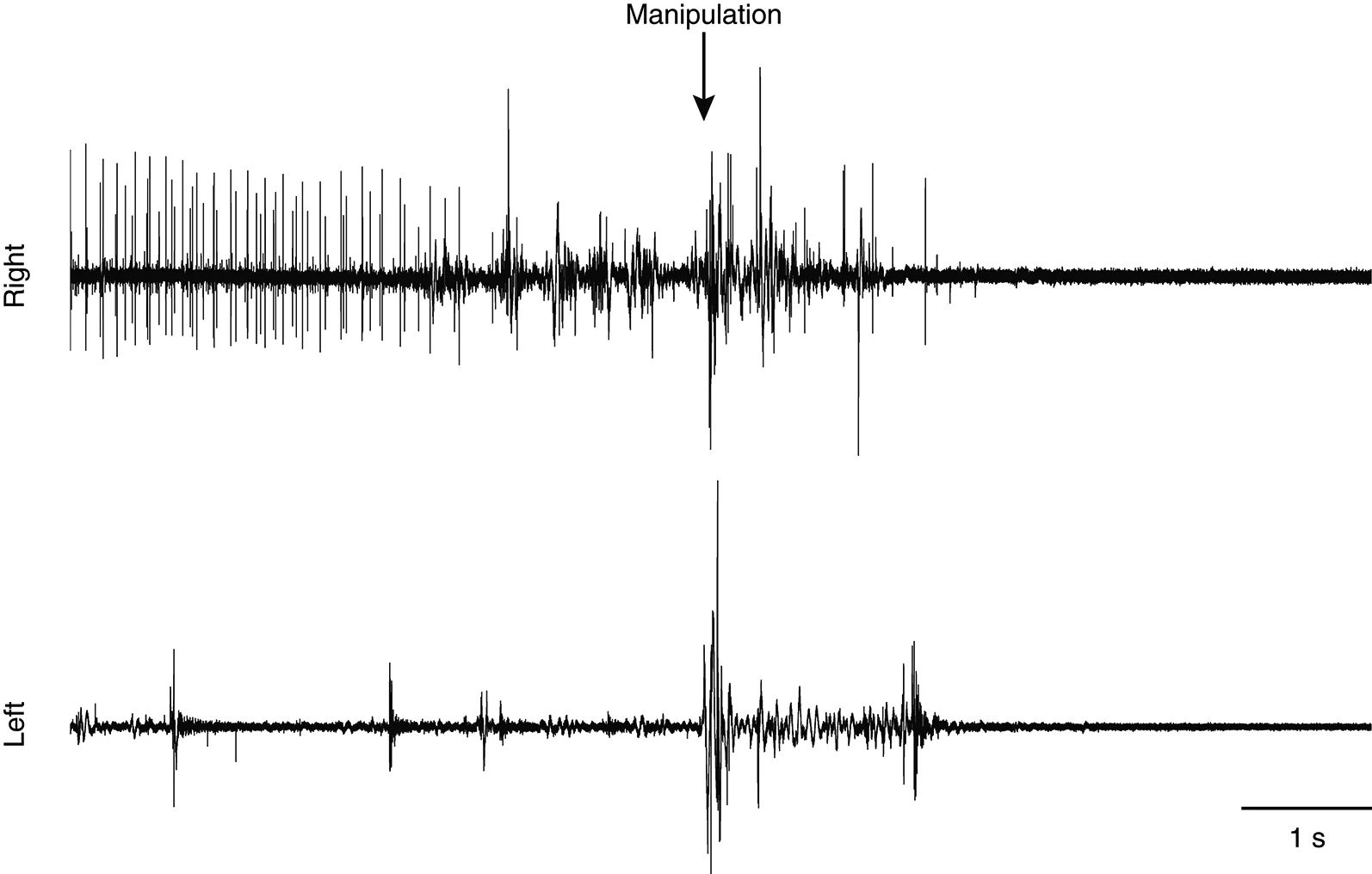
Bilateral recording of the period immediately before and after spinal manipulation (HVLA-SM). The subject is in a left recumbent position with the spine in right rotation. Motoneuron firing can be seen on the right side before HVLA-SM but not after. All other subjects displayed no activity immediately before and after HVLA-SM, i.e., similar to the bottom trace. The signal during and immediately subsequent to the spinal manipulation is most likely caused by movement artifacts

One HVLA-SM was performed per side for each of the 9 subjects. EMG activity was recorded from each of the 16 successfully implanted electrodes during both ipsi- and contralateral HVLA-SM, i.e., a total of 32 recordings. The HVLA-SM was identified from fine-wire EMG signals as shown in Fig. 1 and synchronized with video recordings. In all but one subject there was electrical silence both immediately before and after HVLA-SM. In one subject there was single MU activity during the preceding 5 s before HVLA-SM but not the 5 s after (Fig. 1). In the same subject there was bilateral MU activity both before and after the second HVLA-SM when lying on the opposite side. The thrust EMG burst is interpreted as a mechanical/movement artifact and does not reflect motor unit activity. The intervention procedure lasted less than 10 min (median 5:13 (min:sec), quartile 4:03–7:17, range 3:26–7:57).

### Gross activity from EMG raw signal

A total of four hours of recording time after HVLA-SM was available for analysis (Table 1), 9–19 min (range) per subject during spontaneous sitting and 5–20 min during spontaneous standing. Total gross muscle activity duration was 22% of recording time (range 0–99%) during spontaneous sitting and 56% (range 0–100%) during spontaneous standing. In addition, recorded activity during voluntary contraction ranged from 0.5 to 2.4 min.

**Fig. 2:**
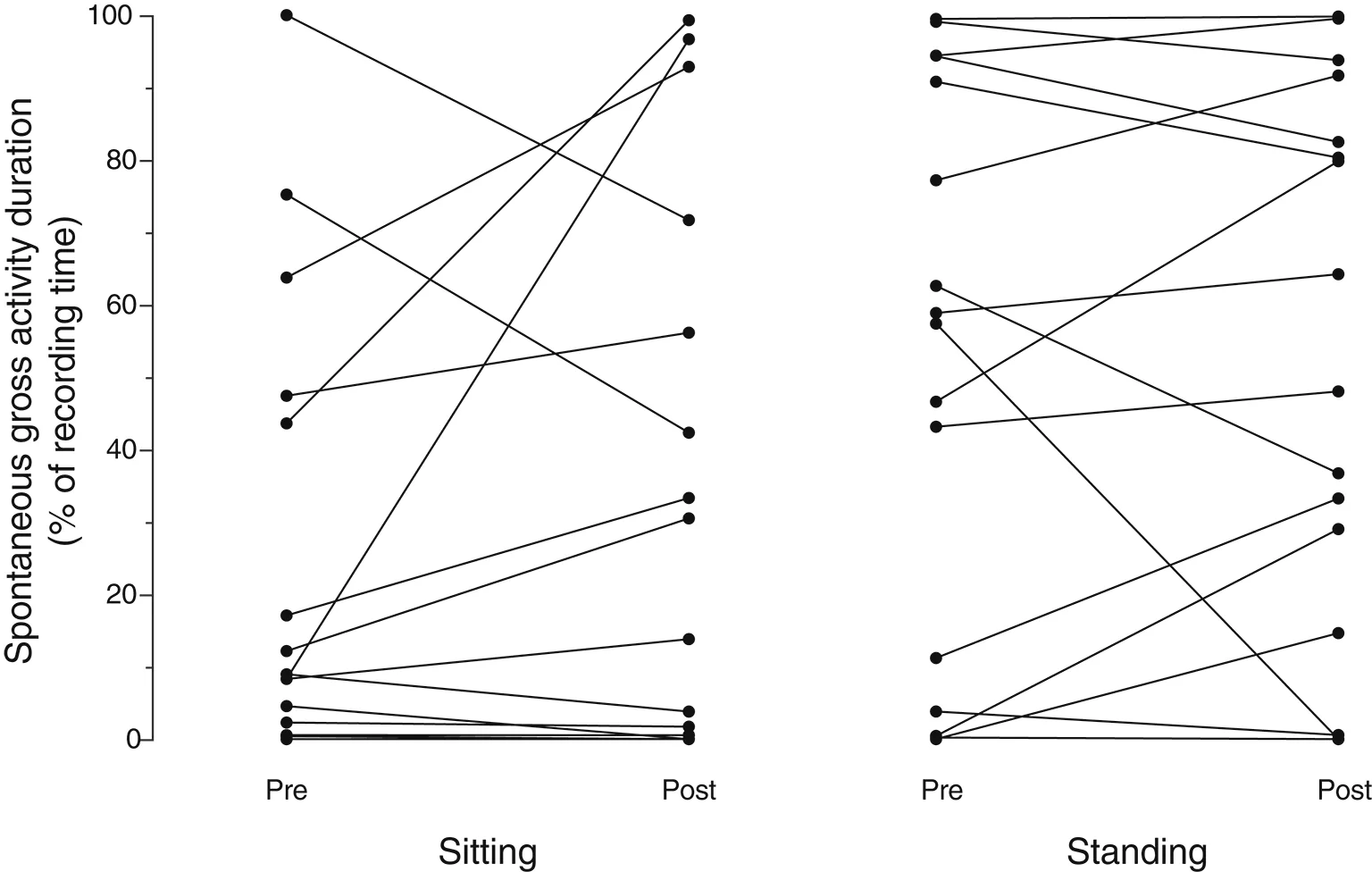
Spontaneous gross EMG activity duration during sitting and standing before [27] and after HVLA-SM. Recordings from the same acute low back pain subject and side are connected by lines. There was no significant change after HVLA-SM

The mixed model used to evaluate contribution of variables that might have influence on gross muscle activity duration (see Methods) did not reveal any significant effects. Similarly, no difference was found between total gross activity duration before [27] and after HVLA-SM. Individual data are shown in Fig. 2.

### Discharge rate

**Fig. 3:**
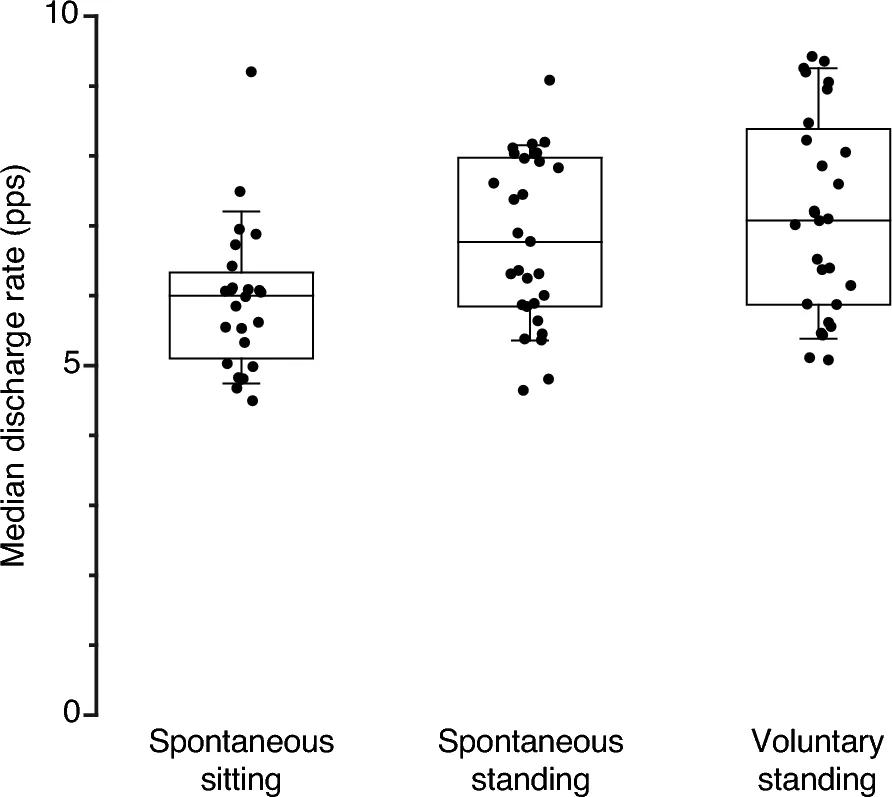
Distribution of median motor unit discharge rates after HVLA-SM. Distribution shown for spontaneous activity during sitting, and for spontaneous and voluntary activity during standing. Boxes show medians and quartiles; whiskers denote 10 and 90 percentiles. Standing and voluntary activity contributed significantly toward higher median discharge rates (Tables 1 and 2)

We have previously described discharge characteristics of 73 units with a total of 113,063 inter spike intervals in the same 9 subjects before HVLA-SM [27]. We were able to discriminate 69 distinct MUs with a total of 59,168 intervals after HVLA-SM (Table 1; Fig. 3). Their median discharge rate was 6.0 pps during spontaneous sitting, 6.8 pps during spontaneous standing, and 7.1 pps during voluntary force production with auditory feedback while standing. The final mixed-model analysis demonstrated significant effects of sitting vs. standing and of spontaneous versus voluntary activity (Table 2). No difference was found between discharge rate before [27] and after HVLA-SM (*P* = 0.53).

**Table 2:** Effects on discharge rate, interspike interval variability, and common drive

Independent variables	Discharge rate (pps)		∆ISI IQR (ms)		CDC	
	Effect size (95% CI)	*P*	Effect size (95% CI)	*P*	Effect size (95% CI)	*P*
Sitting to standing	1.22 (0.75–1.69)	< 0.0001	15.24 (7.83–22.65)	< 0.0001	NS	
Spontaneous to voluntary	0.65 (0.21–1.09)	0.0042	− 20.62 (−14.13–−27.10)	0.0001	NS	
Bilateral vs unilateral MU pair	NS		NS		− 0.149 (− 0.087–− 0.210)	< 0.0001
Change per ms increase in ISI	–		0.29 (0.16–0.42)	< 0.0001	–	
Total model RSquare	0.81		0.60		0.99	

ISI, inter-spike interval. ∆ISI IQR, inter-quartile range for differences between consecutive ISIs. MU, motor unit. CDC, common-drive coefficient. pps, pulses per second. ms, millisecond. CI, confidence interval. NS, not significant and consequently not included in the model

### Interspike interval variability

**Fig. 4:**
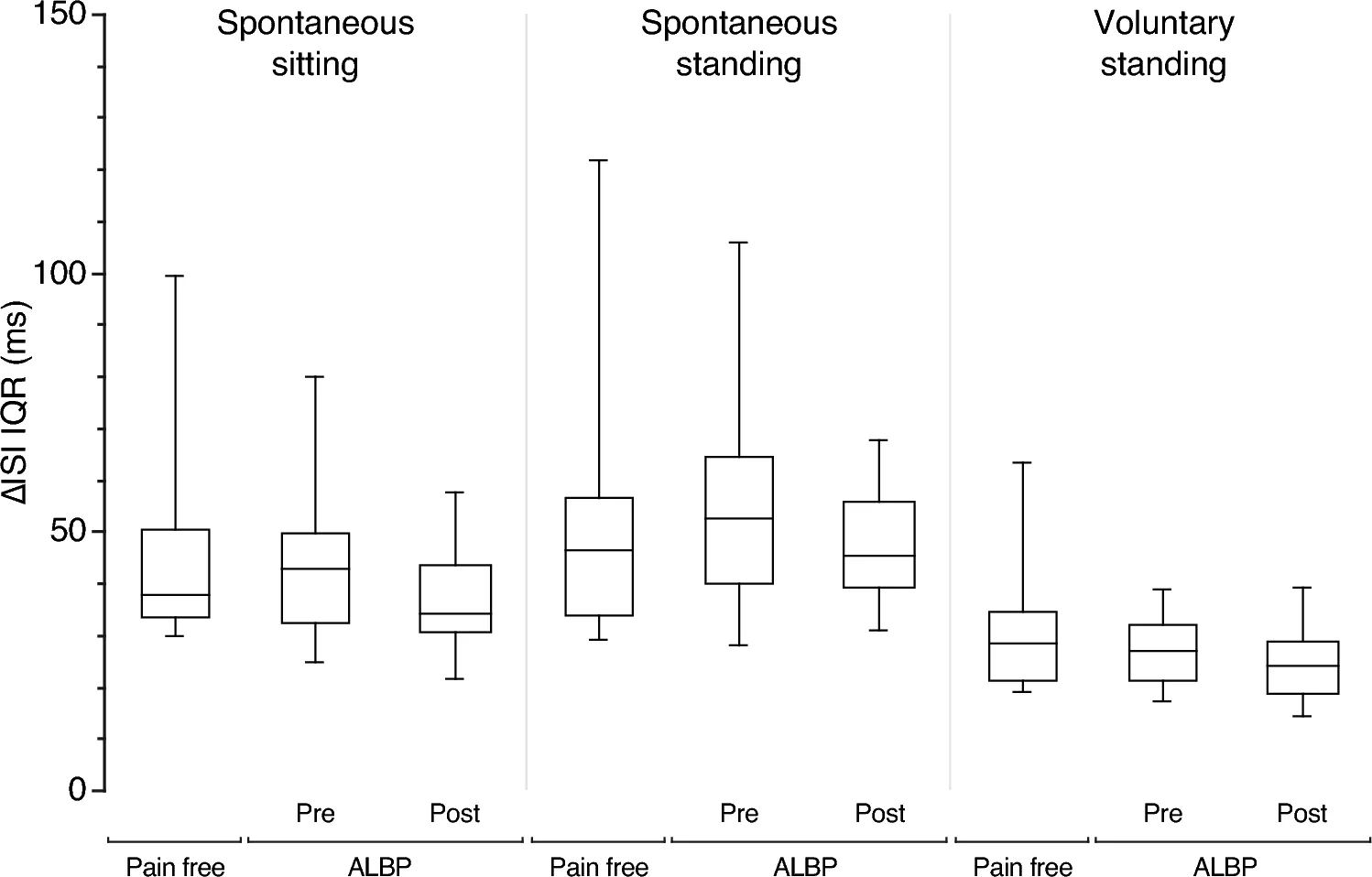
Interspike interval variability (∆ISI IQR) in spontaneous sitting and standing as well as during voluntary standing. Data from acute low back pain (ALBP) subjects before (Pre) [27] and after (Post) spinal manipulation (HVLA-SM); previously published results from 9 healthy subjects with no history of back pain [14] are shown for comparison. Boxes show medians and quartiles; whiskers denote 10 and 90 percentiles. Standing and spontaneous activity contributed significantly toward a higher ∆ISI IQR in the ALBP Post HVLA-SM group (Tables 1 and 2). There was a significant decrease in ∆ISI IQR in the ALBP subject group after HVLA-SM

**Fig. 5:**
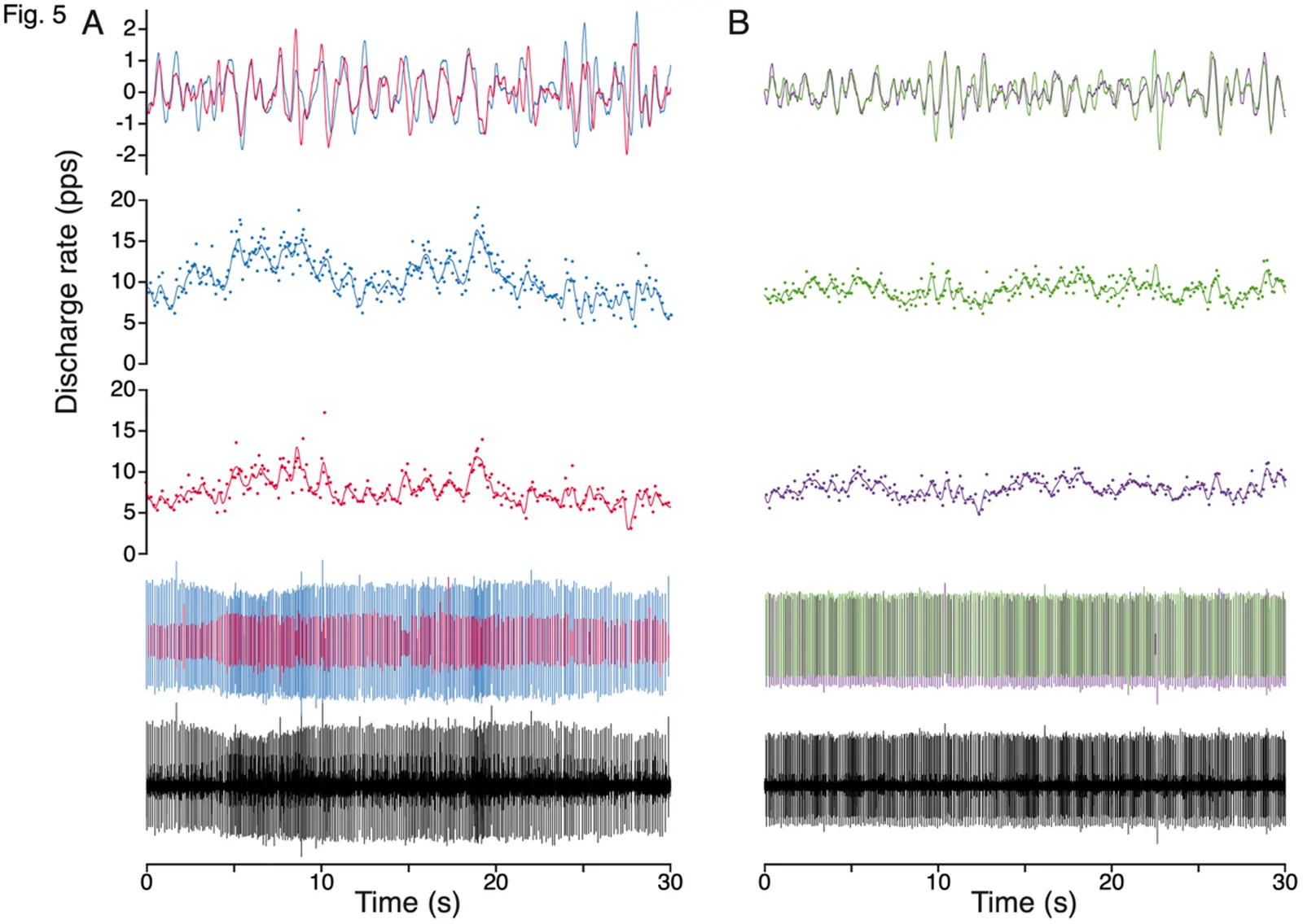
Unilateral recordings from the painful side of an ALBP patient. Concurrently active units are shown during spontaneous standing before (**A**) and after (**B**) HVLA-SM. From bottom to top: Unprocessed analogue signal (black), discriminated MUs (colour coded), superimposed instantaneous and smoothed discharge rates for each colour coded unit, and superimposed smoothed discharge rate traces high-pass filtered at 0.75 Hz to remove mean discharge rates and low-frequency oscillations (cf. Figure 3 in [14]). Note smaller scatter in discharge rate after HVLA-SM, and correlated oscillations indicating a strong common drive in both situations. Different units before and after HVLA-SM

Interval-to-interval variability was assessed for each MU as the interquartile range for differences between successive interspike intervals (ΔISI IQR) (Table 1, Fig. 4) and evaluated with the same mixed model approach. As expected [47], discharge variability was larger with longer ISI (Table 2). ∆ISI IQR also increased by 15.24 ms from sitting to standing and decreased by 20.62 ms from spontaneous to voluntary activity (Table 2). When adjusting for ISI, sitting vs. standing and spontaneous vs. voluntary activity in a mixed-effects model, ∆ISI IQR was reduced by 5.25 ms after HVLA-SM compared to data from the same subjects before manipulation [27] (95% CI 0.68–9.82; *P* = 0.025; RSquare = 0.43 for the total model; Figs. 4 and 5).

### Common drive

**Fig. 6:**
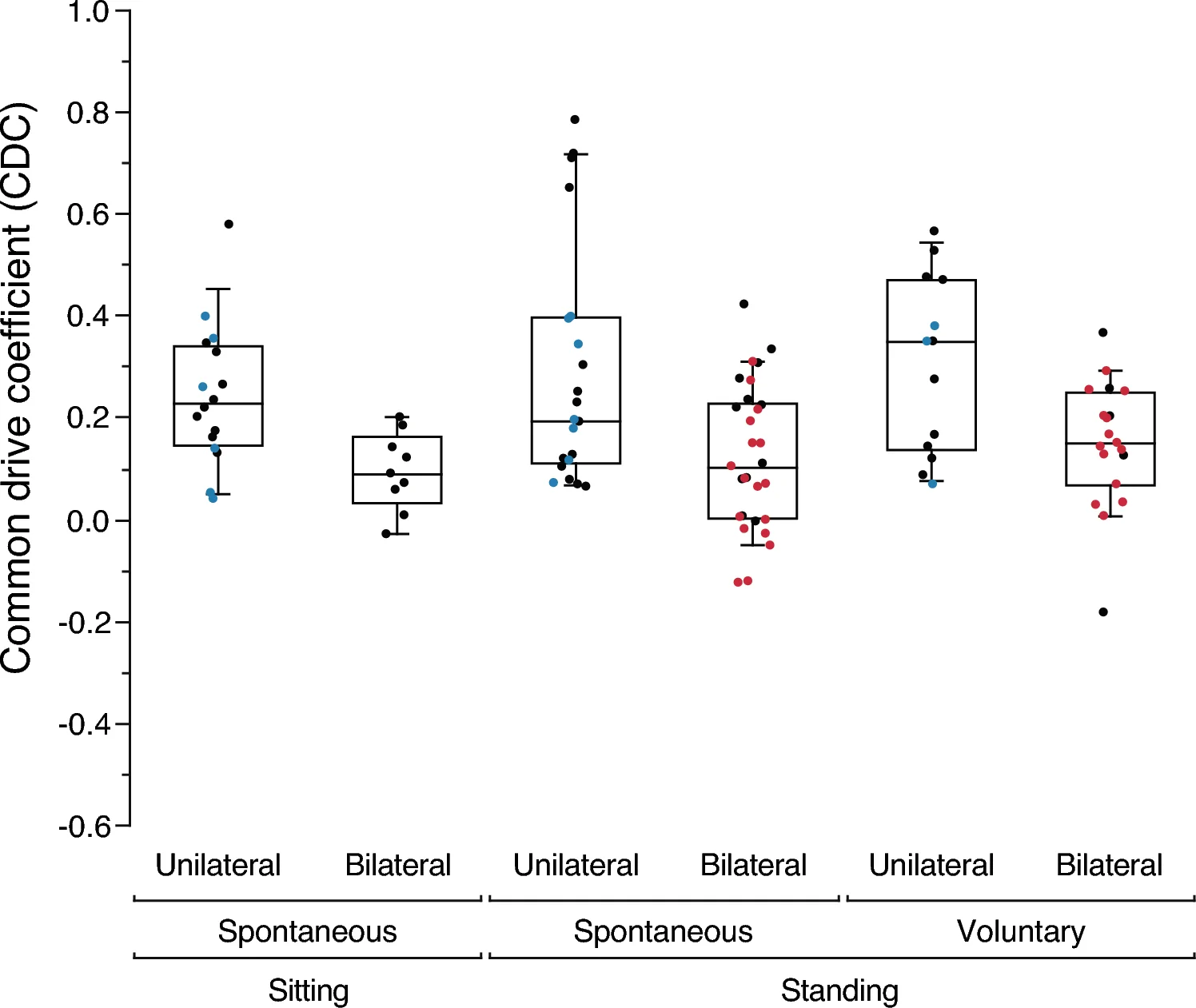
Distribution of common drive coefficients (CDCs) in 49 unilateral and 55 bilateral MU pairs. Boxes show medians and quartiles; whiskers denote 10 and 90 percentiles. MU pairs where one or both units were on a pain free side are shown with red and blue symbols respectively. Unilateral unit pairs contributed significantly toward a higher CDC (Tables 1 and 2)

Concurrently active MUs modulated their discharge rate in synchrony, indicative of a strong common drive (Fig. 5). Common drive after HVLA-SM was assessed quantitatively through CDC analysis of 49 unilateral and 55 bilateral MU pairs from 9 subjects (Table 1; Fig. 6). The mixed model analysis demonstrated only effects of bilateral vs. unilateral MU pairs (Table 2). Similar results were found for spontaneous standing tested separately [27]; here CDC was 0.16 lower for bilateral MU pairs (95% CI 0.07–0.25; *P* = 0.0016; RSquare = 0.40). No difference was found between CDC before [27] and after HVLA-SM (*P* = 0.71).

**Fig. 7:**
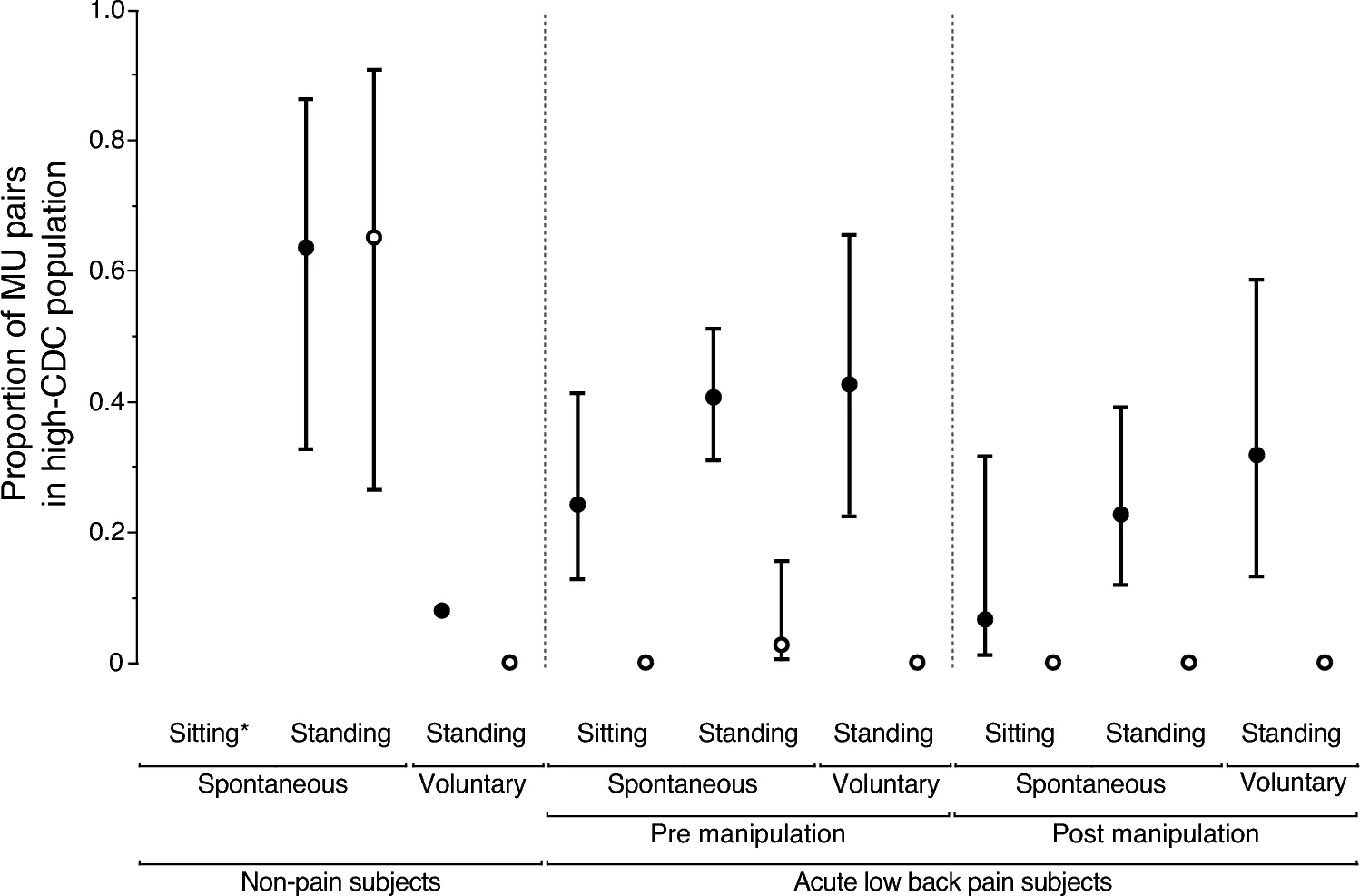
Proportions of common drive coefficients (CDCs) in the higher-CDC sub-distribution, with 95% confidence intervals. Data from the present study shown together with data from the same subjects before HVLA-SM [27]; previously published results from 9 healthy subjects with no history of back pain [14] are shown for comparison. Note demographic mismatch between the ALBP and non-pain subjects Non-overlapping CIs denote significant differences. *Proportions for spontaneous sitting in non-pain subjects could not be computed due to small sample size (unilateral n = 3, bilateral n = 4). Closed symbols denote unilateral MU pairs; open symbols denote bilateral MU pairs

We have previously found both in 9 healthy subjects with no history of back pain [14] and in the current patient population before HVLA-SM [27] that the distribution of LM CDC values can be described as a mixture of two Normal distributions, one for low CDC values and the other for high CDC values (see Fig. 7 in [27]). The proportion of CDC values in the high-CDC subpopulation in pain-free subjects was strikingly similar for unilateral and bilateral MU pairs, in contrast to the current population before HVLA-SM where it was significantly lower for bilateral recordings [27]. Determination of the proportion of MU pairs in the high-CDC subpopulation was therefore also performed in the current population after HVLA-SM. Figure 7 shows the results with 95% CIs, together with previously published data from the 9 pain free subjects for comparison [14, 27]. Similar to before HVLA-SM, the proportion of bilateral MU pairs in the higher-CDC subpopulation was significantly lower than for unilateral MU pairs (non-overlapping 95% CIs). Individual unilateral proportions were neither significantly different from another nor from previously reported unilateral data.

### Self-sustained firing

Conclusive evidence of rotation of MU activity according to our predefined criteria was not found in the present material. MUs did jump to a preferred firing range after recruitment and continued to fire together with already active units, but we were not able to find periods where the already active unit was derecruited while the newly recruited unit maintained its activity.

## Discussion

We have found a reduction in ISI variability after HVLA-SM, whereas total duration of gross EMG activity, median motor-unit discharge rate, and common drive were not changed when compared to findings in the same subjects before HVLA-SM [27]. Notably, common drive for spontaneous standing was still lower in bilateral unit pairs than in unilateral pairs, in contrast to our findings in pain free subjects where the two were not significantly different (Fig. 7; see also Fig. 7 in [27]). Tonic muscle activity showed signs of self-sustained firing, but we could not identify rotation between motor units. No immediate effects on MU activity were evident in the first seconds after HVLA-SM. While HVLA-SM was associated with a reduction in ΔISI IQR (*P* = 0.025), this finding should be considered suggestive rather than definitive given the exploratory nature of the study and the number of parameters tested. For gross activity, discharge rate, and CDC, no large or consistent effects were detected; however, the modest sample size limits our ability to exclude subtle but potentially relevant changes in these variables.

### Implanted electrodes

The present material demonstrates that implanted fine-wire EMG electrodes are suitable for following MU activity in the deep LM before, during, and after spinal manipulation. To be confident of the results of intramuscular EMG it is necessary to confirm electrode placement. By use of CT verification of electrode placement after the subject had moved and the electrodes had settled in the tissues, we have a high degree of confidence that the electrode has indeed recorded from the intended target muscle [27].

Whilst it is possible that the electrode implantation in the LM could have affected EMG activity, the subjects demonstrated similar finger to floor measurements before and after implantation indicating minimal restriction from the electrode. Additionally, the subjects had no complaints of increased or altered pain following electrode implantation or during the experiment. Earlier studies comparing dynamic surface EMG before and after implanting fine-wire electrodes suggested that the procedure has minimal effect on muscle activity [48]. The small volume of subcutaneous lidocaine used was of short duration and unlikely to affect the deep multifidus.

### Immediate effects of spinal manipulation

The surface EMG pattern caused by HVLA-SM has been proposed to be composed of a series of spatially and temporally non-synchronized MUAPs of various origins [25, 49, 50]. However, in our material the HVLA-SM events recorded by the fine-wire EMG electrode were clearly different from MUAPs (Fig. 1). The EMG signal at the moment of HVLA-SM can most probably be interpreted as a movement artifact, and we are therefore hesitant to attribute undue significance to it.

We were unable to demonstrate any clear immediate effect of HVLA-SM on MU activity. Several authors have utilized surface EMG to determine the effect of HVLA-SM on paraspinal muscles [51–53], and the consistent finding in these studies is a reduction in EMG signal immediately following manipulation. In comparison, Herzog [54] reported a more variable response to HVLA-SM in subjects with hypertonic paraspinal thoracic musculature, where the muscles relaxed and surface EMG activity was abolished in some but not all patients [54]. Our inclusion criterion was pain on palpation and not muscle hypertonicity, which may have contributed to a different result as postural muscle activity is not to be expected in the recumbent relaxed position. Furthermore, unlike intramuscular fine-wire EMG, the use of surface EMG does not allow any conclusions to be drawn on the effect of HVLA-SM on the deep-lying lumbar multifidus [23].

While prior intramuscular-EMG work by Colloca et al. [26] finding that spinal manipulative thrusts elicit immediate, transient neuromuscular responses temporally coupled to the applied force (typically occurring within 2.4–18.1 ms), our study focused on the sustained influence of HVLA-SM on spontaneous tonic activity. Our finding that HVLA-SM reduces ISI variability suggests that beyond the immediate reflexogenic ‘burst’ identified in force-coupled studies, the HVLA-SM intervention facilitates a more stable motoneuron output during subsequent postural challenges like standing and sitting. This suggests that the neurophysiological impact of manipulation may involve both a short-lived stimulus–response and a more enduring modulation of postural control mechanisms.

By use of implanted electrodes in anaesthetised pigs, Indahl et al. [55] witnessed a decreased LM EMG response after injecting hypertonic saline into the facet joints. The authors interpreted the response to be caused by stretching of the joint capsule, subsequently exciting inhibitory interneurons resulting in reflex inhibition of motoneurons. Further, they suggested that the beneficial effect of HVLA-SM in some cases may result from stretching of the facet joint capsule thereby inhibiting muscle spasm. A subsequent study performed to confirm these findings was unable to reproduce them [56]. Whilst it is interesting to speculate that facet joint capsule stretch due to injection and manipulation are similar, Herzog [38] demonstrated that a fast treatment thrust induced muscle activation whereas slow force application did not. Likewise, Randoll [57] demonstrated that the ability of HVLA-SM to specifically decrease pain was not seen with only a light mechanical stimulus over the spinous process. Thus, it is reasonable to speculate that individual chiropractors will deliver HVLA-SM at different speeds that may in turn differently influence the physiological response [54].

### Gross-EMG activity and motor-unit discharge rate

The varied gross-EMG response to HVLA-SM found in the literature [51–53] is to a degree consistent with our findings. Gross-EMG activity was increased post-manipulation in some of our subjects and decreased in others, with similarly varied responses in both sitting and standing (Fig. 2). LBP is a complicated multifactorial problem, and it may therefore not be surprising that our subjects displayed different postural strategies. According to Hodges and Tucker [58], pain is not a simple switch causing uniform inhibition or facilitation across the motor system. Instead, it acts like a complex orchestra conductor, selectively modifying the timing and gain of various components (motoneurons, spinal circuits, and cortical input) to achieve the overall goal of protecting the painful part from further injury. This complex, multi-level adaptation may generate the highly variable patterns of movement observed.

It is important to note that the voluntary task relied heavily on real-time auditory and visual biofeedback to match targets. Consequently, the differences observed between the spontaneous and voluntary conditions do not reflect a pure comparison of descending motor commands. Instead, the voluntary condition introduces distinct neurophysiological variables—specifically, heightened cognitive attention and sensorimotor feedback loops—that are known to modulate corticospinal excitability and spinal circuitry during precise motor control [44, 58, 59].

The median motoneuron discharge rate after HVLA-SM was similar to what we have observed in pain-free subjects and in the same study group before manipulation. This may imply that the same output from individual motoneurons is necessary to maintain tonic low muscle force when keeping the body upright in the postures we have studied, regardless of pain.

### Changes in interspike interval variability after spinal manipulation

Our data demonstrated a significant difference in ISI variability, measured as ∆ISI IQR, between sitting and standing as well as between spontaneous and voluntary activity (Table 2). Utilising a repeated-measures model we found a significant reduction in variability following HVLA-SM in our ALBP subjects (cf. Figs. 4 and 5), bringing the distribution closer to that of pain free subjects [14]. The magnitude of this reduction corresponds to almost half of the change in median ∆ISI IQR between spontaneous standing and sitting (Table 1).

The reduced ISI variability after HVLA-SM suggests that the procedure may facilitate a return to a more stable motoneuron output. Increased variability, as measured by the coefficient of variation, has been observed in other studies involving induced muscle pain [28], women with neck pain [29], and stress [30]. While variability may be beneficial in the sensory system for conveying information from highly receptive sensors, variability or neuronal “noise” in the motor system lacks any associated benefits, can result in inconsistent force output [31], and may even cause a reduction in maximum force production [32]. Increased ISI variability could conceivably contribute to the reduced muscle endurance associated with LBP [60, 61] and to the observation that LBP patients move more smoothly following spinal manipulative treatment [62]

Our findings may suggest that spinal manipulation facilitates a return to a more stable motoneuron output to the lumbar multifidus. It can be speculated that the observed changes in variability after HVLA-SM may be caused by changed activity in sensory afferents or suprasegmental pathways. However, activation of motoneuronal plateau potentials could also be an underlying factor for the reduced variability in spiking, due to uncoupling of spike generation from the variability in synaptic input and the shunting effects of increased input conductance rendering synaptic inputs less effective [12, 13].

### Common drive

The strong uni- and bilateral common drive to the LM motoneuron pool previously reported in pain free subjects [14] points to the role of the paraspinal muscles in working as a functional unit to extend and stabilize the spine in the upright position. We have previously reported reduced bilateral common drive in the present group of ALBP patients, suggesting altered bilateral control of the spine [27]. A change to a more coordinated bilateral control after HVLA-SM would support a mechanistic theory of HVLA-SM as influencing sensorimotor integration through suprasegmental pathways [63]. However, this was not found in the present study.

We have previously speculated that a compromised common drive may be caused by pain, alternatively that a less tightly coordinated common drive could predispose for back pain [27]. We suggest that the sustained difference in common drive after HVLA-SM favours the latter. This view is in agreement with a recent randomized controlled trial demonstrated that individuals who already relied more heavily on lumbar proprioception experienced greater pain reduction after a single session of HVLA-SM compared to mobilization or no intervention [64]. This suggests that the postural strategy moderates pain, implying it is a contributing factor rather than a consequence of pain.

### Self-sustained firing

Rotation of MU activity is likely important for stable force production and contributes to distribution of periods of rest and activity for motoneurons and muscle cells [15, 65–68]. Although we found signs of self-sustained activity in motoneurons in our ALBP subjects after HVLA-SM, we were unable to demonstrate rotation of MUs. It could be questioned whether absence of rotation is a factor that maintains or contributes to LBP. We strongly suggest that methods to quantify rotation need to be established in order to determine whether self-sustained firing is impeded during ALBP and if HVLA-SM is able to reverse this.

### Strengths and limitations

The present study exhibits several methodological strengths that enhance its validity and clinical relevance. The use of a pain-free intramuscular EMG technique was well tolerated by participants and permitted full spinal mobility. Extended recording durations provided a rich dataset of MUs and MUAPs, capturing activity across common postural tasks. These inherently valid conditions, combined with a repeated-measures design, allowed participants to serve as their own controls and facilitated longitudinal analysis before and after spinal manipulation, reducing sample size requirements without compromising analytical depth. Data integrity was further supported by MUAP computer classification followed by visual confirmation and manual refinement and the use of long-duration segments for common drive coefficient analysis to minimize selection bias. Standardization of intervention was maintained by employing a single experienced clinician for all spinal manipulations.

Several limitations must be acknowledged. Diagnostic uncertainty persisted due to the absence of validated tests distinguishing discogenic from facet-mediated pain, likely resulting in a heterogeneous pain population with variable neurophysiological responses to HVLA-SM. The intentional use of a single clinician to perform standardized HVLA-SM limits the extrapolation of results to a broader practitioner pool. External validity is limited using a convenience sample drawn from two private outpatient clinics, compounded by an imbalanced sex representation among the participants.

To minimise radiation exposure, electrode placement was not verified by CT scanning after the experiment. Demographic and postural heterogeneity between non-pain and acute low back pain cohorts complicated direct comparisons. Measures of gross-EMG activity through the implanted fine-wire electrodes reflected only local motor unit behaviour rather than whole-muscle function. Furthermore, the lack of validated criteria for detecting plateau potentials or defining motor-unit rotation limited accurate quantification of self-sustained firing.

Furthermore, the within-subject, single-session design introduces several potential confounders that the present study cannot fully distinguish from the effect of the manipulation. These include: (a) electrode settling or progressive changes in recording quality over the 2–4 h experimental session; (b) habituation to the laboratory environment and postural tasks; (c) regression to the mean, particularly if the initial pre-recordings captured a period of peak pain or guarding; and (d) the natural early recovery of acute low back pain, which can remit rapidly. While we attempted to mitigate electrode settling by verifying placement via CT only after the subject had moved, and maintained stable environmental conditions, these alternative explanations remain consistent with the observed reduction in ΔISI IQR. Future studies employing multiple pre-intervention baseline epochs or a separate control group are needed to establish a definitive causal link.

Finally, the modest sample size, although large in this type of experiment, may have precluded detection of subgroup effects. The modest sample size of nine patients is a limitation that necessitates caution. While sufficient for this type of complex, invasive experiment, it means that our findings—particularly the ‘no change’ results for common drive and discharge rate—should be interpreted as an absence of large effects rather than proof of no physiological impact. Similarly, the reduction in ISI variability, although statistically significant in our model, is borderline when considering the family of tests performed and requires confirmation in larger, independent samples.

## Conclusions

Our findings demonstrate that manipulation can influence the stability of individual motoneuron output even when broader coordination patterns remained unchanged. Future research should explore if these subtle neurophysiological changes correlate with long-term clinical recovery. The persistence of reduced bilateral common drive after a single HVLA-SM is consistent with (but does not establish) the hypothesis that altered bilateral coordination represents a pre-existing trait rather than a pain-driven state. Longitudinal data through recovery, or comparison to recovered LBP patients, would be required to discriminate these possibilities. The absence of rotation of activity between motor units may conceivably contribute to the muscle fatigue and reduced endurance characteristic of LBP, though further methodological refinement is needed to quantify this relationship.

The methodology utilized in this study has provided stable long-lasting intramuscular EMG recordings from LM. Spontaneous MU activity was recorded in normal postures reflecting typical LM activity in people suffering from ALBP. In contrast, many studies investigating motoneuron properties examine EMG during strictly controlled voluntary force production [44, 59]. As we have demonstrated in the present study as well as in pain free subjects and in ALBP patients prior to HVLA-SM [14, 27], MU discharge characteristics are different under spontaneous activity compared with voluntary force production. The methodology provides a framework for investigating postural muscle control mechanisms in LBP and highlights the importance of studying spontaneous tonic activity alongside voluntary contractions. This should be taken into account in future studies of postural muscle control mechanisms.

## Data Availability

The datasets used and/or analysed during the current study are available from the corresponding author on reasonable request.

## Abbreviations

ALBP: Acute low back pain
CDC: Common drive coefficient
CI: Confidence interval
CT: Computer tomography
EMG: Electromyography
HVLA-SM: High velocity low amplitude spinal manipulation
IQR: Inter-quartile range
ISI: Interspike interval
LM: Lumbar multifidus
LBP: Low back pain
MRI: Magnetic resonance imaging
MU: Motor unit
MUAP: Motor unit action potential
pps: Pulses per second
